# Introduction of a standardised protocol, including systematic use of tranexamic acid, for management of severe adult trauma patients in a low-resource setting: the MSF experience from Port-au-Prince, Haiti

**DOI:** 10.1186/s12873-019-0266-x

**Published:** 2019-10-18

**Authors:** Alessandro Jachetti, Rose Berly Massénat, Nathalie Edema, Sophia C. Woolley, Guido Benedetti, Rafael Van Den Bergh, Miguel Trelles

**Affiliations:** 1 Médecins Sans Frontières – Operational Centre Brussels – Haiti Mission, Port-au-Prince, Haiti; 2 Emergency Department, Fondazione IRCCS Ca’ Granda Ospedale Maggiore Policlinico, Milan, Italy; 3 Médecins Sans Frontières – Operational Centre Brussels – Operational Research Unit, Brussels, Belgium; 4 Médecins Sans Frontières – Operational Centre Brussels – Surgical and Critical Care Unit, Brussels, Belgium

**Keywords:** Trauma, Tranexamic acid, Emergency room, Low-resource setting, Haiti, Medécins sans Frontières

## Abstract

**Background:**

Bleeding is an important cause of death in trauma victims. In 2010, the CRASH-2 study, a multicentre randomized control trial on the effect of tranexamic acid (TXA) administration to trauma patients with suspected significant bleeding, reported a decreased mortality in randomized patients compared to placebo. Currently, no evidence on the use of TXA in humanitarian, low-resource settings is available. We aimed to measure the hospital outcomes of adult patients with severe traumatic bleeding in the Médecins Sans Frontières Tabarre Trauma Centre in Port-au-Prince, Haiti, before and after the implementation of a Massive Haemorrhage protocol including systematic early administration of TXA.

**Methods:**

Patients admitted over comparable periods of four months (December2015- March2016 and December2016 - March2017) before and after the implementation of the Massive Haemorrhage protocol were investigated. Included patients had blunt or penetrating trauma, a South Africa Triage Score ≥ 7, were aged 18–65 years and were admitted within 3 h from the traumatic event. Measured outcomes were hospital mortality and early mortality rates, in-hospital time to discharge and time to discharge from intensive care unit.

**Results:**

One-hundred and sixteen patients met inclusion criteria. Patients treated after the introduction of the Massive Haemorrhage protocol had about 70% less chance of death during hospitalization compared to the group “before” (adjusted odds ratio 0.3, 95%confidence interval 0.1–0.8). They also had a significantly shorter hospital length of stay (*p* = 0.02).

**Conclusions:**

Implementing a Massive Haemorrhage protocol including early administration of TXA was associated with the reduced mortality and hospital stay of severe adult blunt and penetrating trauma patients in a context with poor resources and limited availability of blood products.

## Background

### Introduction

Tranexamic acid (TXA) is a synthetic lysin-analog that inhibits fibrinolysis by blocking the lysine-binding sites on plasminogen. The use of TXA was first described in the early 1970s for the control of bleeding associated with urinary tract surgery [1] and for haemorrhage associated with dental extraction in patients with haemophilia [2]. In the 1990s, TXA use was expanded to the treatment of hyper-fibrinolysis associated with cardiopulmonary bypass, in which it was proved to reduce blood loss and the need for transfusion [3–5]. Further studies on gynaecological bleeding [6, 7] and elective orthopaedic and abdominal surgery found that TXA reduced the need for transfusions [8, 9]. The number of studies on the use of TXA in elective surgery has increased since the 2000s, providing adequate evidence of its safety profile [10, 11]. Recent studies have confirmed how TXA can reduce overall mortality, blood loss, and mortality related to bleeding in many types of elective surgery, without observable adverse effects [12–18].

Bleeding is an important cause of death after trauma [19, 20]. In 2010, the Clinical Randomization of an Antifibrinolytic in Significant Haemorrhage (CRASH-2) study, a multicentre randomized clinical trial on the effect of TXA administration to trauma patients with suspected significant bleeding, reported a decreased mortality in patients randomized to TXA as compared to a placebo [21]. Since the CRASH-2 study, the number of studies related to the use of TXA in trauma setting has increased. Many recent studies and systematic reviews confirmed the findings of the CRASH-2 study, thus prompting many hospitals in different settings to adopt TXA in their massive haemorrhage control protocols [22–24]. The use of TXA was further explored in paediatric trauma [25, 26] and prehospital settings [27, 28], showing reduced mortality and no side effects. TXA was also tested in military settings in 2012, with a first research known as the MATTERs study [29], followed by others [30–32], with results for combat casualties also holding promise. In 2016, the European Guidelines for Management of Major Bleeding recommended the use of TXA in trauma [33].

### Trauma induced coagulopathy

Trauma is one of the major causes of morbidity and mortality at global level, especially among the young. Approximately 1.2 million trauma-related deaths occur annually, while the numbers of hospitalized patients (around 24 million) and patients who require extra-hospital medical care (around 84 million) are increasing, which adversely impacts healthcare resources. Haemorrhage is the most important cause of death in severe trauma victims, through two main mechanisms: fatal haemorrhage from vascular injury resulting in haemorrhagic shock, and secondary bleeding coagulopathy. The latter represents a pathological entity by itself, and is also termed Trauma Induced Coagulopathy (TIC) or Acute Trauma Coagulopathy (ATC). About 25–35% of trauma patients develop a clinically detectable and demonstrable coagulopathy even before hospital arrival, and they have a risk of death within 24 h that is up to eightfold higher than patients without signs of coagulopathy. The mechanisms underlying this process have not yet been fully clarified [34, 35]. Shock and systemic hypoperfusion could be triggers [36]. Considering hemodilution, recent studies only show significant association between pre-hospital fluid administration and onset of coagulopathy for infusion volumes greater than 2200 ml [37–39]. The effect of hypothermia on coagulant proteases is minimal for temperatures above 33 °C but a certain level of correlation with the onset of coagulopathy was observed [40–43]. Acidosis has a dose-dependent effect on the coagulation function. However, the normalization of pH do not result in reversing the coagulopathy, and more complex mechanisms underlying TIC may exist [44–46]. Each hospital should develop, implement and adhere to a management protocol, adapted to the local setup [29, 47, 48].

### General setting

Haiti is a Caribbean country, with a population of more than 10 million inhabitants, approximately 25% of whom live in the metropolitan area of the capital Port-au-Prince [49]. The country suffers from poor health indicators, lacks adequate resources and infrastructure, and struggles to meet the population’s basic medical needs and to provide adequate services, such as maternal healthcare and acute trauma care. While a segment of the population can access private health services in Haiti or overseas, healthcare is generally difficult to access for a large proportion of Haitians. Medical facilities are often understaffed and lack funding to cover for medical supplies and operating costs.

The Médecins Sans Frontières (MSF) Tabarre hospital in Port-au-Prince, Haiti, is a referral Trauma Centre operating in a low-resource context, where bleeding and TIC are among the leading causes of death for victims of accidental and violent trauma. Blood transfusion is not readily available in this context, and the early administration of TXA may therefore contribute to saving lives and optimizing resources. This study aimed to assess the clinical and hospital outcomes of adult patients with severe traumatic bleeding in the MSF Tabarre Trauma Centre before and after the implementation of a new Massive Haemorrhage protocol, which included early administration of TXA.

## Methods

### MSF Tabarre trauma Centre

In Tabarre, a densely populated suburb of Port-au-Prince, MSF has been running a 121-bed hospital providing surgery and trauma-related care since 2012. In 2015, the Emergency Department (ED) was attended by more than 13,000 patients, and the hospital performed more than 8200 surgical interventions for 6400 patients [50]. Physiotherapy, mental health and social support were also offered to victims of trauma. The hospital was built to respond to the lack of adequate treatment for surgical and traumatic emergencies in the capital city after the 2010 earthquake.

### The south African triage score

**Fig. 1:**
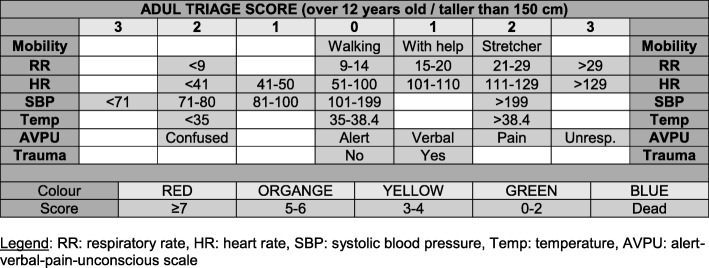
SATS score (adapted from South African Triage Group 2008©). Legend: RR: respiratory rate, HR: heart rate, SBP: systolic blood pressure, Temp: temperature, AVPU: alert-verbal-pain-unconscious scale

The South African Triage Score (SATS) was developed in 2006 for use in both pre- and in-hospital emergency units throughout South Africa. It aimed to address the increasing burden of severe emergency cases, medical staff shortages, and limited resources. The score was validated in emergency care by studies in South Africa [51, 52] and in various settings such as Malawi [53], Botswana [54], Ireland [55], and Pakistan [56]. As one of the few triage scores validated specifically in poorly resourced settings [57], MSF applied the SATS since the opening of Tabarre hospital. The SATS score is based on vital signs, mobility and level of consciousness (see Fig. 1) and due to its characteristics, its reliability and capacity to anticipate in-hospital mortality [58, 59] were chosen as measure of injury severity. The ISS (Injury Severity Score), the most widespread measure of injury severity in high-resource settings, was impractical in the setting of this study due to its dependence on anatomical scoring evaluation using parameters that could not be measured in this setting, and due to its poor reliability due to the different backgrounds of the MSF surgeons.

### Massive Haemorrhage protocol

In late 2014, the MSF Critical Care Working Group elaborated the Massive Haemorrhage protocol (see Additional file 1). This protocol describes the actions to be taken in case of suspected haemorrhages: maintaining normothermia and haemostasis, resuscitation with permissive hypotension (systolic blood pressure 90 mmHg), early transfusion, and rapid access to the Operating Theatre (OT). The protocol also recommends the early intravenous (IV) administration of 1 g of TXA in 10 min, if possible and maximally within 3 h after injury, followed by 1 g IV of TXA in 10 min, 3 h after the 1st bolus. The protocol was introduced in Tabarre in December 2016 as part of a strategy aiming to maximise hospital resources and to strengthen the skills of the staff. The number of doctors working at the ED was doubled in April 2016 and all staff (doctors, nurses, auxiliary service) received additional training on trauma management and the use of TXA in October and November 2016.

### Study design and population

This was a descriptive retrospective ‘before and after’ un-paired study measuring the clinical outcomes of adult patients with severe traumatic bleeding in the MSF Tabarre Trauma Centre. The outcomes of patients accessing the hospital over 4 months before the implementation of the Massive Haemorrhage protocol (December 2015 to March 2016, group “before”) were compared with the outcomes of patients accessing the hospital over the same period one year after (December 2016 to March 2017, group “after”). This was a retrospective analysis of routinely collected data. Included cases were adults aged 18–65 years, with blunt or penetrating trauma and a SATS score ≥ 7, arriving at the ED within 3 h from trauma and being classified as “red” codes.

### Data handling and analysis

Data was sourced from the routinely implemented monitoring and surveillance databases (line-lists) and patient files at MSF Tabarre Trauma Centre. Exposure variables for the groups “before” and “after” included demographic (age), clinical (type of trauma, delay since trauma, SATS score) and case management (type of referral, time of access, Massive Haemorrhage protocol) characteristics of patients accessing the MSF Tabarre Trauma Centre (ED, OT, in-patient department and intensive care unit). Included patients were categorised as self-referred (yes or no), arrived ≤/> 1 h from trauma, having penetrating or blunt trauma, having multiple injuries, undergoing surgery (yes or no). Outcomes included intra-hospital mortality (yes or no), early mortality (yes or no, considered as death within 48 h from arrival), number of days spent in the hospital (in-patient department and intensive care unit). Data was extracted and imported into a dedicated structured electronic spreadsheet (Excel 2010, Microsoft Corp, Redmond, WA, USA). All exposures and mortality outcomes were categorical and summarised as counts and proportions. Duration of hospitalisation was a numerical continuous outcome, and was summarized as median and inter-quartile range (IQR). Associations between exposures and the two groups (“before” and “after”) were measured by Fisher’s exact test. Associations between the duration of hospitalization and the two groups (“before” and “after”) were measured by Wilcoxon rank sum test, first including the entire study population and secondarily considering only patients with favourable outcome (alive at exit) from hospital. The strength of association among exposures, the two study groups (“before” and “after”), the mortality causes and outcome (alive or dead at exit from hospital) were measured by bivariate and multivariate logistic regression and were expressed as odds ratios (ORs) and 95% Confidence Intervals (CI). *P*-values< 0.05 were considered statistically significant. Analysis was performed with STATA/IC Version 14.1. (StatCorp, College Station, TX, USA).

This study fulfilled the exemption criteria set by the MSF Ethics Review Board (ERB) for a posteriori analyses of routinely collected data and was approved by the Haitian Ethics Review Board.

## Results

Overall, 116 patients met the inclusion criteria: 52 from the group “before” the introduction of the Massive Haemorrhage protocol, and 64 from the group “after”. Table 1 shows a cascade of the included patients per group. The median age for the group “before” was 28 years [IQR 23–36], while it was 32 years [IQR 26–41] for the group “after”. The female/male ratio was 0.1 and 0.2 respectively in the two groups. In the “before” group, 6 patients had blunt trauma as consequence of traffic accident and 46 had a penetrating trauma (40 gunshot wounds and 6 knife wounds). In the “after” group, 7 patients had blunt trauma (6 traffic accidents and 1 fall) and 57 had a penetrating trauma (46 gunshot wounds and 11 knife wounds). The two groups were similar in terms of characteristics of trauma (Table 2). However, patients treated after the introduction of the Massive Haemorrhage protocol had a significantly longer delay between the time of trauma and the time of arrival (*p* < 0.001) and they underwent less surgical treatment (*p* < 0.01) than the others.

**Table 1:** Number of patients included in the study by group (before and after)

	Group Before	Group After
	12, 2015–03, 2016	12, 2016–03, 2017
Accessed Emergency Department	4559	5330
SATS score ≥ 7	1119	1528
Age 18–65 years	885	1170
Blunt/penetrant trauma	193	204
Arrival ≤3 h from trauma	153	126
Received Massive Hemorrhage protocol	n.a.	70
Red cases^a^ retained for analysis	52	64

SATS: South Africa Triage Scale - ^a^according to SATS score

**Table 2:** Trauma characteristics and case management of patients by group (before and after)

	Group Before	Group After	*P*-value*
	12, 2015–03, 2016	12, 2016–03, 2017	
	*n* = 52	*n* = 64	
	count (%)	count (%)	
Auto referral	34 (65.4)	40 (62.5)	0.85
Referred	18 (34.6)	24 (37.5)	
Arrived ≤1 h from trauma	31 (59.6)	15 (23.4)	< 0.001
Arrived > 1 h from trauma	21 (40.4)	49 (76.6)	
Penetrant trauma	37 (71.2)	33 (51.6)	0.04
Blunt trauma	15 (28.8)	31 (48.4)	
Multiple injuries	28 (53.9)	32 (50)	0.71
No multiple injuries	24 (46.1)	32 (50)	
Undergone surgery	45 (91.8)	43 (67.2)	< 0.01
No surgery	4 (8.2)	21 (32.8)	
*Fisher exact test			
All patients	Group Before	Group After	*P*-value**
	12, 2015–03, 2016	12, 2016–03, 2017	
	n = 52	n = 64	
	median [IQR]	median [IQR]	
Time in-patient department	5 [4–9]	4.5 [3–8]	0.18
Time intensive care	4 [1–6]	4 [2–6]	0.40
Overall time in hospital	8 [4–11]	6 [3–9]	0.23
**Wilcoxon rank sum test			
Alive only	Group Before	Group After	*P*-value**
	12, 2015–03, 2016	12, 2016–03, 2017	
	n = 52	n = 64	
	median [IQR]	median [IQR]	
Time in-patient department	5 [4–9]	4.5 [3–8]	0.18
Time intensive care	4 [1–6]	5 [2–6]	0.52
Overall time in hospital	8 [6–12]	6 [4–9]	0.02^a^

*Fisher Exact Test **Wilcoxon rank sum test; ^a^significantly associated

The two groups showed similar hospital lengths of stay in the in-patient department and intensive care unit. When excluding patients with unfavourable outcome (i.e. event of death), patients treated after the introduction of the Massive Haemorrhage protocol had a significantly shorter hospital length of stay: 6 days [IQR 4–9] vs 8 [6–12] (*p* = 0.02).

In the group “before”, 18 deaths were observed, 16 for bleeding (89%, OR 0.5 95%CI 0.0–7.6), 1 for sepsis and 1 for severe traumatic brain injury. In the group “after”, 10 deaths were observed, 9 for bleeding (90%, OR 0.3, 95%CI 0.0–23) and 1 for severe brain injury associated with blunt trauma. Out of all unfavourable outcomes, early mortality was recorded in 13 cases for the group “before” (72%) and in 8 cases for the group “after” (80%), OR 1.5 95%CI 0.2–20. Table 3 describes factors associated with unfavourable outcome in the entire study population. Patients exposed to the Massive Haemorrhage protocol (group “after”) had 70% lower odds of death during hospitalization compared to the group “before” (adjusted OR 0.3, 95%CI 0.1–0.8). In a multivariate model, cases of blunt trauma were associated with the death of patients as compared to other kinds of trauma (adjusted OR 9.2, 95%CI 2.4–35.4).

**Table 3:** Factors associated with favorable or unfavorable outcome of patients

	Alive	Dead	Bivariate logistic regression	Multivariate logistic regression
	*n* = 88	*n* = 28	crude OR (95%CI)	adjusted OR (95%CI)
	count (%)	count (%)		
Group Before	34 (65.4)	18 (34.6)	–	
Group After	54 (84.4)	10 (15.6)	0.35 (0.14–0.85)	0.32 (0.12–0.84) ^a^
Arrived ≤1 h from trauma	31 (67.4)	15 (32.6)	–	
Arrived > 1 h from trauma	57 (81.4)	13 (18.6)	0.47 (0.20–1.12)	
Penetrant trauma	84 (80.8)	20 (19.2)	–	
Blunt trauma	4 (33.3)	8 (66.7)	8.4 (2.30–30.68)	9.15 (2.36–35.43) ^a^
Multiple injuries	47 (78.3)	13 (21.7)	–	
No multiple injuries	41 (73.2)	15 (26.8)	1.32 (0.56–3.10)	
Undergone surgery	65 (73.9)	23 (26.1)	–	
No surgery	22 (88)	3 (12)	0.39 (0.11–1.41)	

^a^significantly associated

## Discussion

To the best of our knowledge, this is the first study investigating the standardized approach and the systematic use of TXA in a referral third level trauma centre operating in a context of urban violence, lack of pre-hospital medical care, poor resources and with no capacity of massive systematic blood transfusion. The MSF Tabarre Trauma Centre, with its high caseload of severe traumatic bleeding injuries, allowed to evaluate the outcomes related to the implementation of a Massive Haemorrhage protocol including the early administration of TXA. The study showed favourable outcomes for patients after the implementation of the protocol.

Available evidence on the use of TXA mostly comes from high-resource settings. Studies from low-resource contexts (e.g. the CRASH-2 [21] study) typically considered settings where transfusion was available.

TXA dosage after trauma is 1 g IV in 10 min, followed by another gram in 8 h, as available in literature [21, 33]. Such a dosage was validated for elective surgery [60–62] and at the time of writing no attempts to evaluate other dosages have been made in civilian trauma settings. Some studies in orthopaedic surgery demonstrated the efficacy of a bolus of TXA in different doses (from 15 mg/Kg to 30 mg/Kg) instead of continuous infusion [63–66].

In a military context, the MATTERs study [29] proposed a single IV gram of TXA to be infused as soon as possible after trauma and a second gram within 24 h; however, that analysis was not stratified by dose.

The MSF protocol included 1 g IV in 10 min upon arrival and 1 g after 3 h, but no similar indications were found in the literature. Other humanitarian non-governmental organizations have proposed the use of TXA in their trainings but no protocol is available. The dose proposed by MSF reflects the difficulties to have 8 h of infusion and adequate devices in its operational contexts.

Considering all the above, the high caseload, and the high mortality rate of severe bleeding from blunt or penetrating trauma in the MSF Tabarre Trauma Centre, where massive transfusion is difficult, justified this evaluation.

The capacity, size and equipment of the ED of Tabarre Trauma Centre underwent virtually no change between December 2015 and March 2016 (group “before”). In April 2016, MSF doubled the number of Emergency doctors on day and night shifts, but did not change its approaches and protocols until the introduction of the Massive Haemorrhage protocol. Even with two ER doctor on shift, no difference in the admission time for severe patients was observed. No changes in the OT setting were implemented in the study period. Training was performed throughout 2016 by organizing scientific days and bedside coaching. Specific trainings on the protocol and the use of TXA in trauma were performed just before the implementation of the protocol.

No secondary or adverse effects after infusion of TXA were registered during this study, confirming the TXA safety profile. Our findings will support the implementation of the protocol, which is now promoted across MSF surgical and trauma projects.

### Limitations

The “before” and “after” groups showed a few differences: patients treated after the protocol underwent less surgery than the “before” group patients. The higher prevalence of blunt trauma, which does not necessarily require surgical care, could justify this difference. Also, the “after” group showed a longer delay between the event of trauma and arrival at ED: as the increased delay would be expected to carry unfavourable effects, the positive results in the “after” group are likely an underestimation. Concerning all other exposures (demographic, clinical and case management) the two groups were similar.

Two comparable periods of four months were considered sufficiently long to mitigate the punctual effect of any operational change at OT level (e.g. the turnover of colleagues with different background, the exposure of staff to a single lecture on trauma management).

It was not possible to account for the effect of co-morbidities on patients’ outcome because these characteristics were not routinely recorded. However, the specific trauma target of the hospital and the median young age of the patients likely limit their relevance to our analysis.

Another limitation was the poor consistency of the registered delay between time of arrival at ED and case management, which was not available in most of the cases and was therefore excluded from analysis as an exposure.

In the “after” cohort, prehospital times of arrival to the ED after the injury had occurred were higher than in the “before” group, so reduced mortality could also be related to a higher mortality in the pre-hospital setting, which was not possible to account for in this study. Due to lack of Emergency Medical Services and due to the particular setting, obtaining data on pre-hospital care and mortality was impossible, but to our knowledge no changes in the prehospital handling between the two study periods occurred.

In the “after” cohort we observed a reduced surgery rate (92% versus 67%). It can lead to think that patient in the second cohort were less severe and it could explain the reduced mortality in this group, by the way according to severity score we were treating patient with the same severity and with the same probability of death. Moreover, the death rate for bleeding was similar in both groups, so we expect our cohorts to be similar in terms of population.

Finally, a certain number of eligible patients in the “after” group were not treated following the protocol. This was internally investigated by matching the marked therapies with the TXA vial consumption at ED, and by interviewing the doctors. The investigation demonstrated that the protocol was always respected and TXA administered in most cases, but was not recorded in the patient file. All such patients were excluded from analysis.

## Conclusions

This study showed how a standardized approach for the care of severe adult blunt and penetrating trauma patients, including early TXA administration, was associated with reduced mortality in consequence of bleeding in a context with poor resources and not readily available massive blood transfusions. Results were in line with the literature on the use of TXA in severe trauma. Data showed that the dose of TXA used in the MSF protocol was effective, similarly to other dosages from developed countries. The difficulties to put in place massive transfusions in a low-resource setting did not appear to affect the capacity of TXA to contribute to preventing and mitigating TIC, reducing mortality of severely injured trauma patients. More evidence is now required from similar contexts in order to better investigate the dosage and effect of TXA in severe trauma and to compensate for the limits of a monocentric study. Overall, the favourable outcomes of patients exposed to treatment in the “after” group should not be considered an effect of TXA alone. It was rather an effect of the implementation of the entire Massive Haemorrhage protocol (including the training of staff, an improved operational circuit, the availability of a trauma specialist, and the general standardization of the approach by the protocol). Moreover, TXA is not to be considered as a substitute of blood transfusions, which remain key to manage severe trauma. TXA is no “panacea” but an option to reduce the death toll in severe trauma. Its effect is logically more evident wherever adequate blood transfusion is not readily available, thus suggesting its relevance in low-resource settings. The implementation of this protocol was also associated with a reduced hospital stay, carrying additional benefits for patients and the hospital.

This study contributed to the evidence for using TXA as a component of trauma management packages. Together with its use in pre-hospital care (ambulances and first aid posts, as supported by recent literature [28–30]), the systematic use of TXA associated with adequate staff training and the standardisation of trauma care could mark a difference in the outcome of patients in low-resourced and precarious humanitarian contexts. Future studies in low-resources context should be performed to collect data and evaluate the impact of our actions. Standardized protocols should reduce mortality and improve efficiency of our medical project. Other actions and studies might be focus on the improvement of the of pre-hospital care system as a strategy to reduce the mortality of this patient population.

## Supplementary information

**Additional file 1.** Massive Haemorrhage protocol.

## Data Availability

Data is property of MSF – Operational Centre Brussels. The datasets used and analysed during the current study are available from the corresponding author on reasonable request and upon approval of MSF – Operational Centre Brussels.

## Abbreviations

ATC: Acute Trauma Coagulopathy
CRASH: Clinical Randomization of an Antifibrinolytic in Significant Haemorrhage
ED: Emergency Department
ICU: Intensive Care Unit
ISS: Injury Severity Score
LIC: Low Income Country
LuxOR: Luxembourg Operational Research
MSF: Médecins Sans Frontières
OCB: Operational Centre Brussels
OT: Operating Theatre
SATS: South African Triage Score
TIC: Trauma Induced Coagulopathy
TXA: Tranexamic Acid
WHO: World Health Organisation
