# Supplementary material for: Introduction of a standardised protocol, including systematic use of tranexamic acid, for management of severe adult trauma patients in a low-resource setting: the MSF experience from Port-au-Prince, Haiti
Source: BMC Emerg Med. 2019 Oct 18;19:56. doi: 10.1186/s12873-019-0266-x (PMC6798378; doi:10.1186/s12873-019-0266-x)
Supplement: Supplementary file 1 — Additional file 1. Massive Haemorrhage protocol. [file 12873_2019_266_MOESM1_ESM.docx]

**ANNEX 1: Massive Haemorrhage protocol**

Below the massive haemorrhage protocol approved and implemented in Tabarre Trauma Centre during the period of the study. It is in French, the language used by the medical and paramedical staff in the hospital, in order that everyone can understand and follow the rules. It contains some inclusion criteria for the study, the initial approach and the four pillar of the massive haemorrhage treatment: Tranexamic Acid, Haemostasis, Fluids and temperature control.

**Legends**:

Inclusion criteria:

- Age ≥15 years old
- Blunt thoracic or abdominal trauma or penetrant trauma
- Vascular haemorrhagic lesion on limbs
- SATS triage score > 5
- Delay of the trauma < 3 hours

Approach to follow:

- Get 2 IV lines (collect blood samples for cross check, RBC count and glycemia)
- High flow O_2_
- Monitor vital functions
- Use pressing bag for infusions
- Blood transfusion if available

**Tranexamic Acid**: 1^st^ bolus immediate 1 g on 10 minutes IV, 2^nd^ bolus after 3 hours 1 g on 10 minutes IV

**Haemostasis** is priority (medication, pelvic binding, tourniquet, suture) -> Immediate surgery even if unstable patient

**Fluids replacement**: pressure objective 90 mmHg systolic pressure -> Ringer Lactate or NaCl 0.9% 500 mL to repeat. Blood transfusions when possible.

**Temperature management**: stop AC, remove wet clothes, dry the patient, warm cover, warm fluids

Fluids:

- NaCl 0.9 or Ringer Lactate 3 times blood loss
- Blood: 1,3 times blood loss
